# Can we optimize locations of hospitals by minimizing the number of patients at risk?

**DOI:** 10.1186/s12913-023-09375-x

**Published:** 2023-04-29

**Authors:** Pasi Fränti, Radu Mariescu-Istodor, Awais Akram, Markku Satokangas, Eeva Reissell

**Affiliations:** 1 School of Computing, University of Eastern Finland, P.O. Box 111, 80101 Joensuu, Finland; 2 Karelia University of Applied Sciences, 80200 Joensuu, Finland; 3 Finnish Institute for Health and Welfare (THL), P.O. Box 30, 00271 Helsinki, Finland

**Keywords:** Health care information systems, Facility optimization, Clustering, Myocardial infarction

## Abstract

**Background:**

To reduce risk of death in acute ST-segment elevation myocardial infraction (STEMI), patients must reach a percutaneous coronary intervention (PCI) within 120 min from the start of symptoms. Current hospital locations represent choices made long since and may not provide the best possibilities for optimal care of STEMI patients. Open questions are: (1) how the hospital locations could be better optimized to reduce the number of patients residing over 90 min from PCI capable hospitals, and (2) how this would affect other factors like average travel time.

**Methods:**

We formulated the research question as a facility optimization problem, which was solved by clustering method using road network and efficient travel time estimation based on overhead graph. The method was implemented as an interactive web tool and tested using nationwide health care register data collected during 2015–2018 in Finland.

**Results:**

The results show that the number of patients at risk for not receiving optimal care could theoretically be reduced significantly from 5 to 1%. However, this would be achieved at the cost of increasing average travel time from 35 to 49 min. By minimizing average travel time, the clustering would result in better locations leading to a slight decrease in travel time (34 min) with only 3% patients at risk.

**Conclusions:**

The results showed that minimizing the number of patients at risk alone can significantly improve this single factor but, at the same time, increase the average burden of others. A more appropriate optimization should consider more factors. We also note that the hospitals serve also for other operators than STEMI patients. Although optimization of the entire health care system is a very complex optimization problems goal, it should be the aim of future research.

## Background

Although the number of patients with ischemic heart disease has decreased in Finland cardiovascular disease remains the most common cause of death [1]. From the health care service system point of view, patients with ST segment elevation myocardial infarction (STEMI) type of cardiovascular disease present challenges as they need to be treated within a given time limit in a facility with capabilities for invasive treatment or face potentially fatal consequences [2–4]. Patients living closer to a percutaneous coronary intervention (PCI) capable cardiac unit (henceforth PCI cardiac unit) have higher chance of survival than those who live far away [5]. Current health care system is under pressure to cut costs and optimize the health care services better. This may lead to reducing the number of hospitals including those that provide acute treatment for myocardial infarction patients.

The optimization of distance from place of residence to appropriate care is critical when a delay means loss of function or even death [4]. This time dependency is apparent in vascular emergencies such as acute coronary syndrome, and particularly in STEMI, where the electrocardiogram findings show specific signs of imminent heart muscle necrosis. The guidelines for STEMI treatment currently call for invasive PCI within 120 min from symptom onset [4, 6]. These lifesaving procedures can only be performed in dedicated PCI cardiac units by experienced specialized cardiologists.

In Finland, PCI cardiac units (*n* = 22) exist in larger hospitals of all mainland hospital districts, but only the five university hospitals have capability to provide PCIs on a 24/7 basis. Other hospitals, health centers, and ambulance services can provide thrombolysis when primary PCI is not immediately available. However, pre-hospital diagnosis (e.g., obtained by utilizing telemedicine) and early transfer to PCI is the preferred option as it reduces delay and results in better outcomes [4, 7]. The locations of PCI capable hospitals are relevant for care of other urgent medical conditions as well and can be considered to represent the “backbone” of optimal acute hospital network.

Studies focusing on prehospital delays have assumed that health care systems are expected to treat patients with PCI within 90 min from the first medical contact when initial point of care is at a PCI cardiac unit [5]. Other travel times to PCI cardiac units (such as 60 min) have also been used [8]. The definition of the threshold for transport time may vary when in-facility delays (door-to-balloon-time) are taken into consideration [2] yet it remains a significant determinant of patient outcomes.

**Fig. 1:**
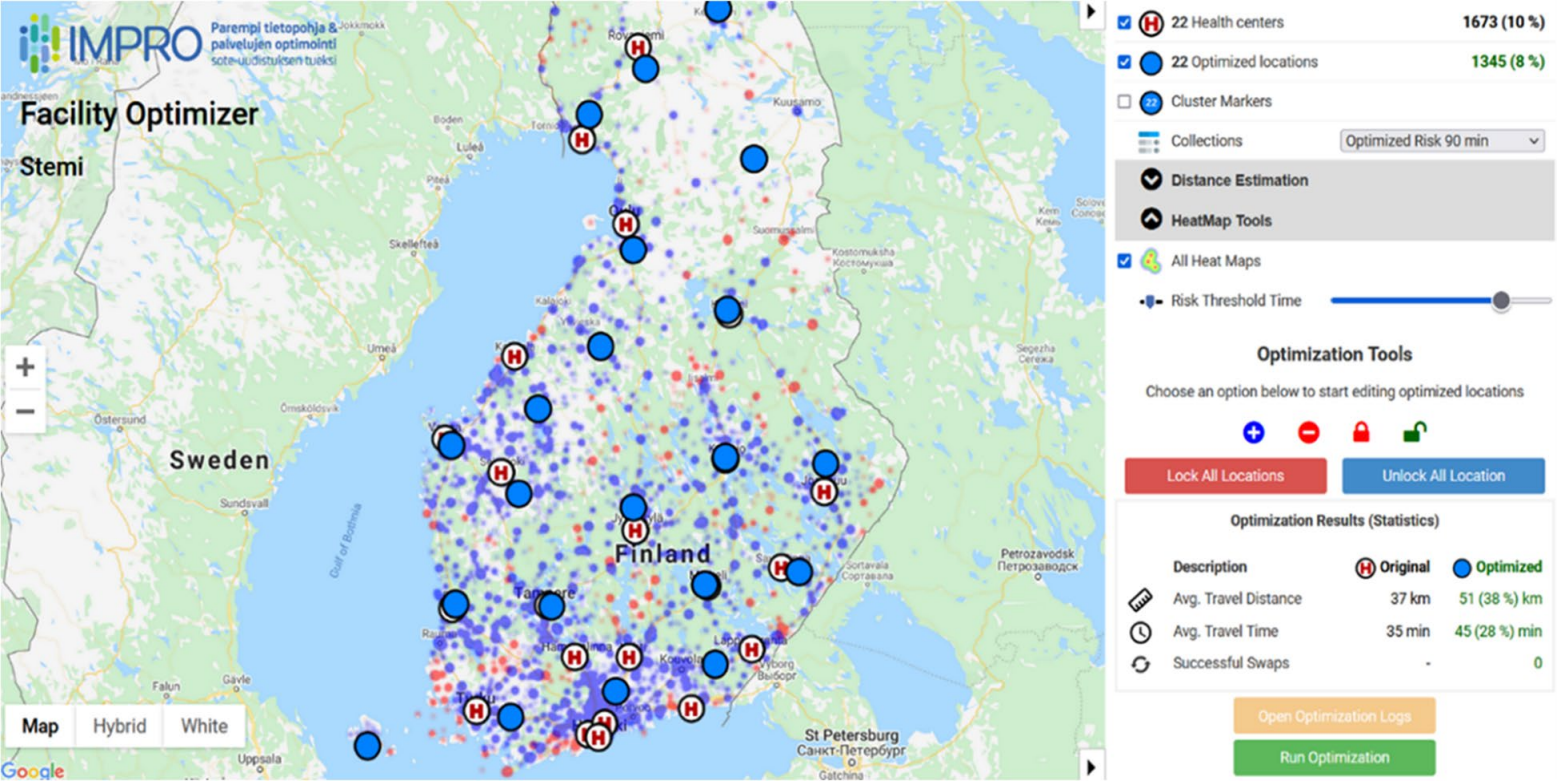
Average travel time from postal code areas to both current and the optimized locations of cardiac units capable of percutaneous coronary intervention (named health centers in the Web-tool)

In this paper, we perform theoretical study how the location of the hospitals would change if they were optimized by minimizing the patients at risk of residing over 90 min from the PCI capable hospital in 2015–2018. We assess optimized hospital locations with an interactive web tool built for this purpose, that optimizes the location by clustering algorithm with real patient data and their location. The optimization result is shown visually on map using the Web-tool described in [9], see Fig. 1.

## Methods

### Data

To analyze a specific group with critical time-dependency, we identified Finnish population with diagnosed ST-elevation myocardial infarctions in 2015–2018 from Finnish Care Register for Health Care. This register is maintained by Finnish Institute of Health and Welfare and includes practically all hospital discharges from both public and private hospitals. STEMIs were defined as hospitalizations with ICD-10 codes I21.0-I21.3. Thus, we excluded other myocardial infarctions requiring less urgent invasive care (classified either as a non-ST-elevation infarction or of an unspecified type). Every individual with at least single hospitalization for STEMI in 2015–2018 was included, 17,563 in total. Those with location information were selected; eventually forming a study population of 17,346 adults.

For every individual in our study population, we obtained postal code area of residence using the personalized ID codes provided by the respective authorities. There are 3038 postal codes in Finland which are larger in sparsely populated areas. To have more accurate home locations of the individuals, we applied a 1 km × 1 km population density grid information from the National Land Survey of Finland and converted the postal code areas to a set of randomized GPS coordinates as follows. We first calculated weighted average of the center points of all grid cells located within a single postal code area. We then allocated number of individuals with STEMI to the GPS coordinates for their respective postal code areas.

Secondary and tertiary care hospitals with PCI cardiac units (*n* = 22) were identified with their corresponding coordinates based on the addresses. In the Web-tool, these units are named *health centers*, as the tool is designed to be general and not limited only to STEMI patients. We also identified the five university hospitals as they have 24/7 capability for providing PCIs.

**Fig. 2:**
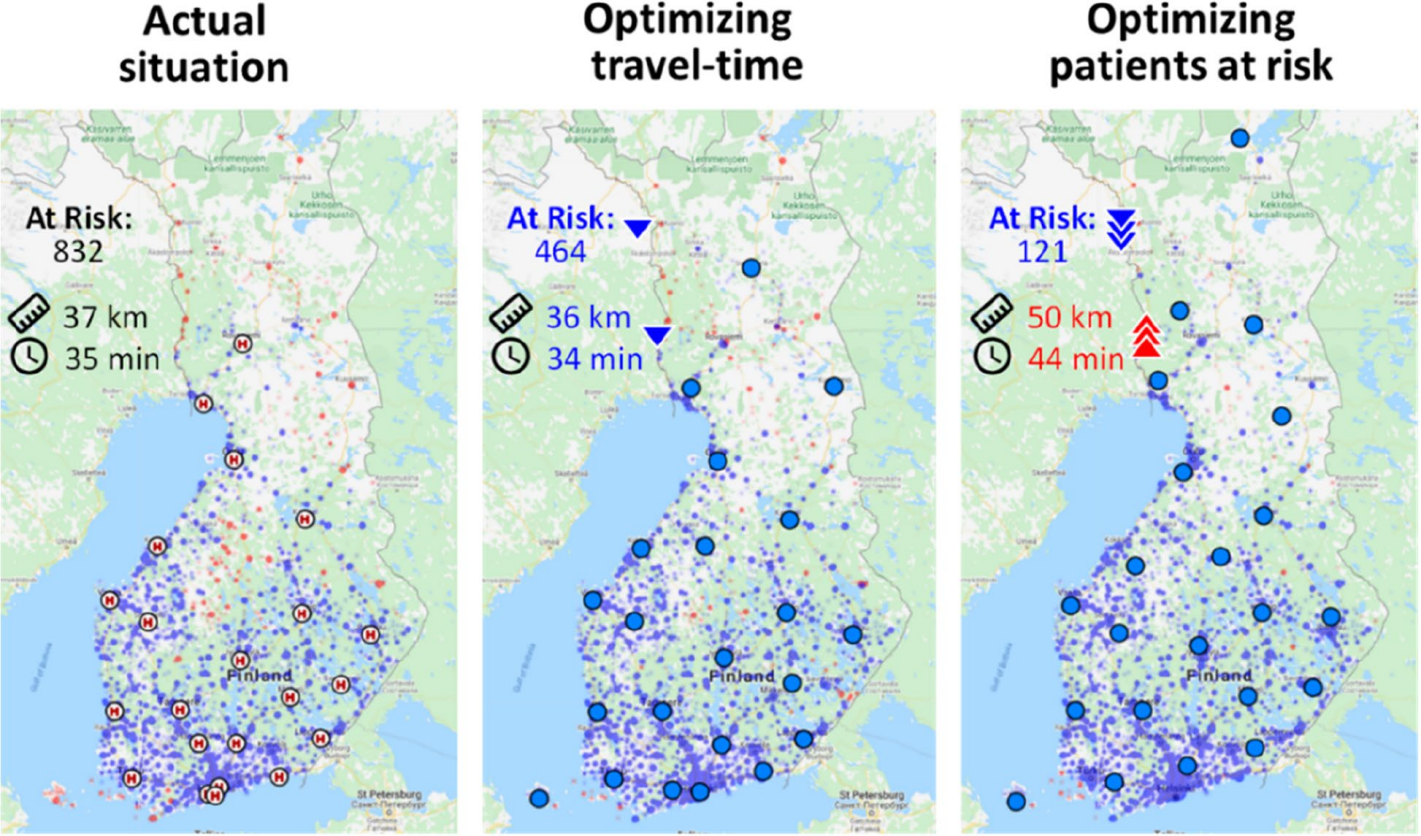
Choice of the cost function has significant impact on the optimization result. Three hospital allocations are shown: the current (left), optimized for minimal travel-time (middle), and optimized for minimum number of patients at risk (right)

Travel times between GPS coordinates of postal code areas and PCI cardiac units were calculated applying open street data. We define individuals with travel time of 90 min or more to the nearest PCI cardiac unit at risk for not receiving PCI in case of STEMI (henceforth *patients at risk*). These individuals are presented in the Web tool with red color, while those whose travel time remain under the threshold of 90 min are depicted blue; see Fig. 2.

### Clustering method

**Fig. 3:**
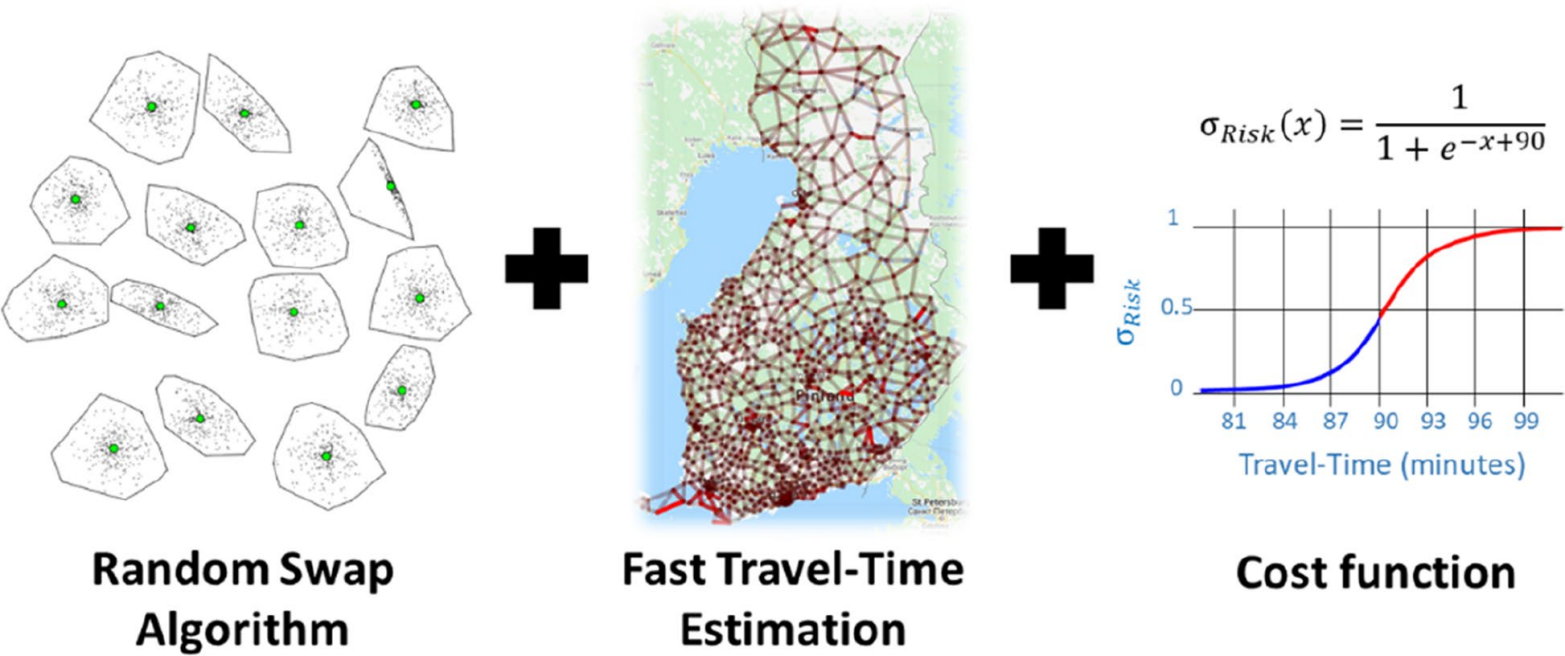
Main components of the clustering include the clustering algorithm, travel time estimation, and the objective (cost function) for the clustering

The optimization is defined as a clustering problem where the input is the home locations of the patients, and the output is the locations of the optimized health centres. The process includes three main components (Fig. 3): clustering algorithm, travel time estimation, and mathematical function that provides the objective for the clustering algorithm.

*K-means* is by far the most common clustering algorithm and would be applicable as such since the data is numeric. Furthermore, the data contains a reasonably small number of clusters (22 at most) of which most are not isolated from the others. Thus, the data would fit rather well for k-means especially if repeated multiple times [10]. However, we do not want to compromise the quality and use improved variant called *random swap*, which is shown to find the best clustering structure even in cases when k-means would fail [11]. High-speed scalable variant of random swap is also available if faster processing time would be needed [12].

While the choice of the algorithm matters for the precision, the choice of the cost function is much more important for the application. We consider two choices: (1) minimizing for travel time, and (2) minimizing for the patients at risk. They are mathematically defined as follows. Optimizing for travel time is simply a linear function which increases with the time. The further the patient is from the hospital, the higher the travel time and the higher its cost in the model.

Minimizing the patients at risk is a bit trickier. In principle, a simple thresholding would assign cost 0 for every patient reaching the hospital within the limit (90 min), and cost 1 for others. However, binary threshold tends to reduce the flexibility of k-means type of algorithms and often lead to inferior optimization result [13]. For this reason, we soften the threshold by using Sigmoid function. The effect is the same as with the linear function: shorter travel times have smaller cost, but there is rapid increase around the threshold value. The benefit of the smoother curve is to avoid possible pitfalls of the algorithm in the optimization.

**Fig. 4:**
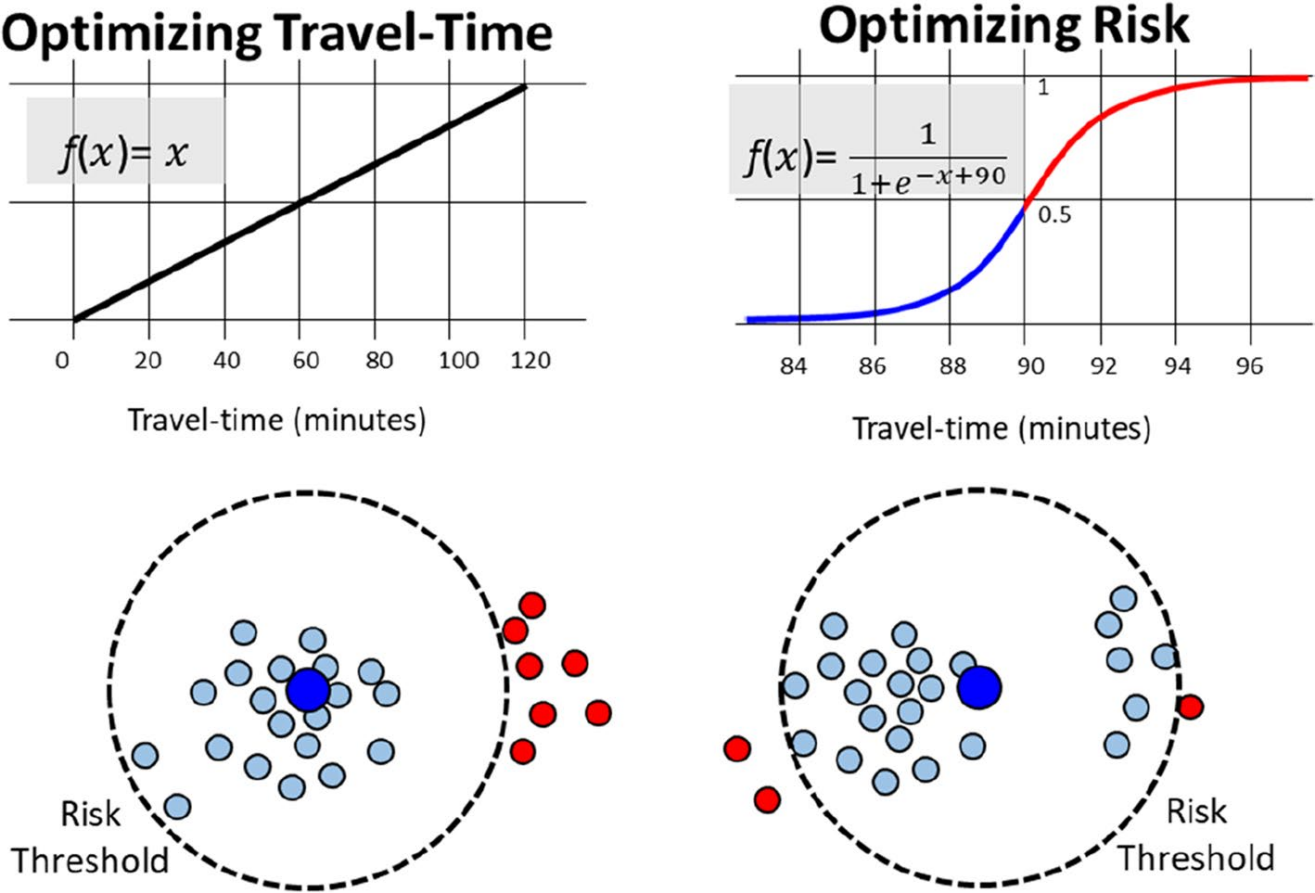
Two cost functions implementing the two different objectives: linear function minimizing travel time (left), and Sigmoid function minimizing patients at risk

The effect of these two cost functions is demonstrated in Fig. 4. In case of Euclidean distance, optimal location for the centre would be the geometric average of all patients which happens to be in the middle of the blue points. The same typically happens when optimizing for travel time as it is directly dependent on the distance. Optimizing for risk, however, tend to move the hospital locations closer to the border of the blue points to reach more of the red points within the time limit.

### Travel time estimation

**Fig. 5:**
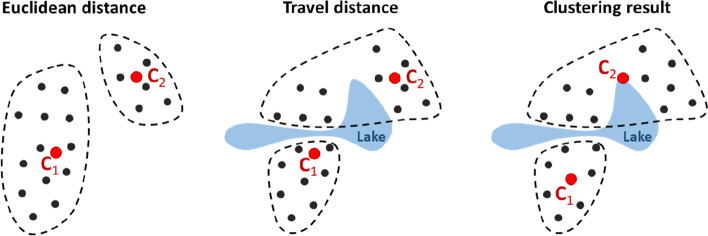
The effect of the distance function on the clustering: Euclidean distance (left), nearest centre according to travel distance (middle), effect on the final clustering (right)

Standard k-means operates with numerical data uses Euclidean distance. However, we use travel distance calculated using road network because simple Euclidean (Bird’s) distance can cause major inaccuracies. It may work well in flat areas with extensive road network [14] but large errors were reported in [15] of about 30% with *SiunSote* data in North Karelia, Finland, in the region of many lakes and rivers. Travel time is then estimated from the travel distance. The use of travel distance instead of Euclidean can also have significant impact on the clustering, see Fig. 5. Other affecting factors are speed limitations and traffic congestions.

The drawback of travel distance is that it is time consuming to compute. The shortest path itself can be easily calculated using road network in linear time but the optimization process may require millions of such operations and the location of the centres are dynamically changing during the process. Fortunately, we do not need to know the actual path but only its length, and more importantly, the length of every path does not need to be exact. It is enough we have their expected length with reasonable accuracy, but we want to have it fast.

To keep the optimization efficient, we therefore use the overhead graph method in [15] to provide rapid estimation for both travel-distance and travel-time. This method has two stages. First, a pre-processed graph is built using the data (patient) locations and the road network to identify most important traffic points, which will be the nodes of the graph. Second, rapid calculation of any travel time (or distance) during the optimization process is done using a look-up table and simple multiplication of the Euclidean distance and an overhead (detour index) provided by the graph.

**Fig. 6:**
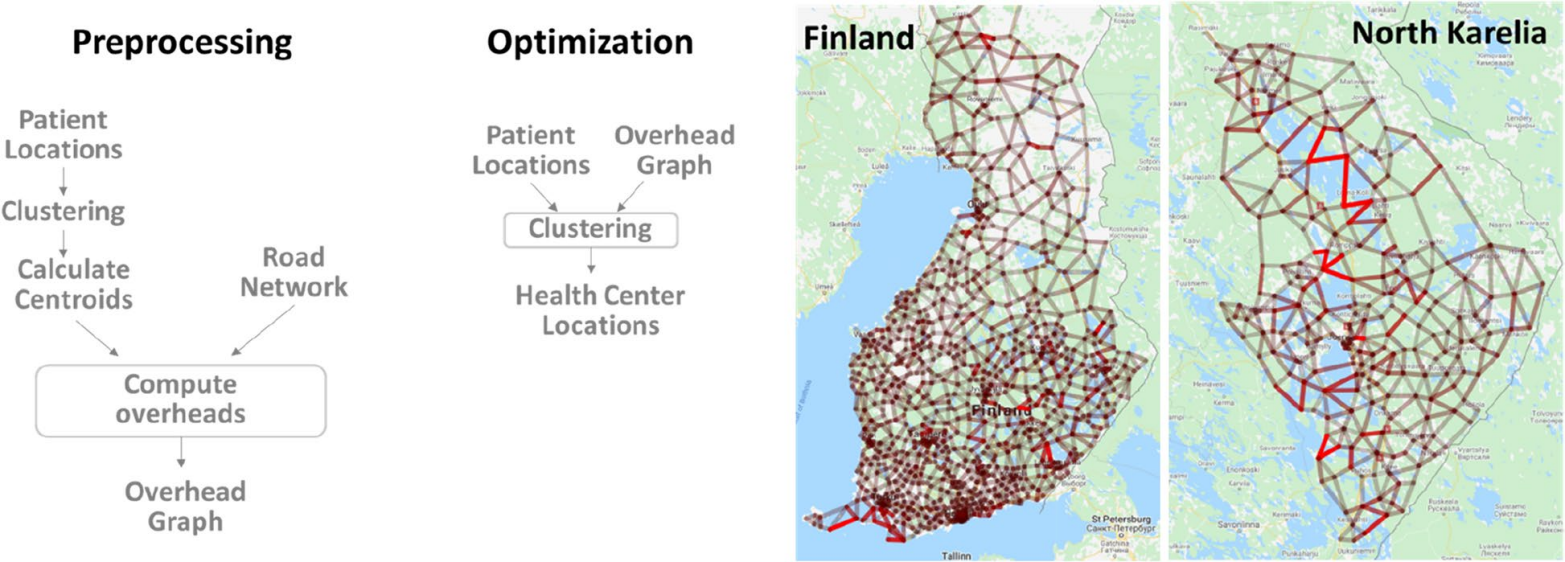
Fast travel time estimation using overhead graph [15]. The two-stage process is shown (left), and two example graphs (right) optimized for Finland (1024 nodes) and for North Karelia region (256 nodes)

The effect of the graph is that the processing time of the optimization is reduced from 1.2 h to only 2.9 s per iteration (up to 10,000) in case of *SiunSote* data in North Karelia using a graph with 512 nodes. The huge speed-up is achieved at the cost of 2% inaccuracy in the distance estimation using memory for 512 × 512 = 0.25 MB look-up for the graph [15]. The process and two sample graphs are shown in Fig. 6.

### Implementation

**Fig. 7:**
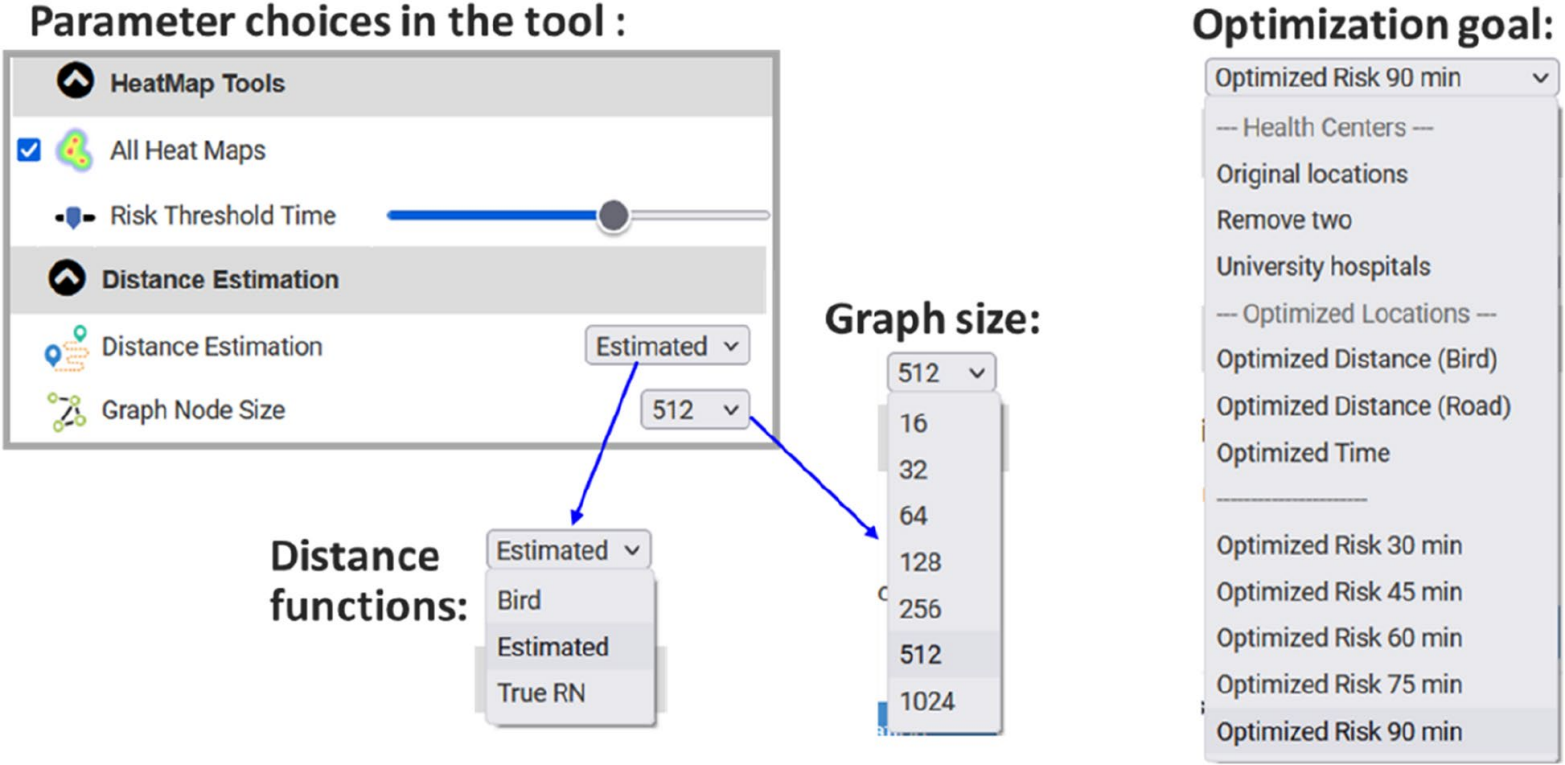
Control parameters for the optimization include risk threshold, distance function, and graph size. Pre-calculations selections are also listed in the optimization goals for faster analysis

The optimization is implemented as a Web tool. The data is pre-calculated and stored within the system, and all the reported combination in this paper have been pre-calculated. The system allows testing also new parameter combinations; see Fig. 7. These include different risk thresholds (30, 45, 60, 75, 90 min), different optimization functions (Euclidean distance, travel distance, travel time, patients at risk), different methods for distance estimation (Euclidean, estimated by the overhead graph, actual travel distance), different graph sizes (16, 32, 64, 128, 256, 512, 1024). The system also allows user to select the number of iterations (50, 100, 2000, 10000) with reporting the estimated processing times varying from 20 s (50 iterations) to 20 min (10000 iterations).

The number of health centres can also be changed either by adding new ones on map, or by removing any existing centre. The location of any centre can be manually changed by drag-and-drop. User can lock certain health centres and optimize only the location of the others. The optimization always starts from the current set of locations shown on map after the manual operations. The algorithm is not sensitive to the initialization and only the locking and the number of centres has significant effect to the outcome of the optimization.

The solutions are visualized on map by showing either the original locations or optimization locations (or both), possibility to show coloured points (heat map) so that red dot indicates patients at risk. Numerical summary is provided by reporting various statistics including the number of patients, the number of patients at risk, average travel time and distance. These are reported as averages of all population, and for each unit separately. However, the visualisation on the map is the easiest way to analyse the optimization result.

The front end of the Web system is implemented by HTML and CSS (styling) and Javascript (interactive elements). The pre-optimized centre locations are stored in MariaDB database, which is a free and open-source community fork of MySQL. Server-side backend is mostly implemented by with PHP but also some parts with Java, Python and few critical parts in C including travel time estimations and the core clustering functions. The system runs on a Centos 7 Linux server with 16 Xeon W-2255 processing cores an 512 GB memory. It is accessible by all stakeholders via passwords.

## Results

Table 1 summarizes the main results of the optimization. The numbers are the averages over all patient locations. We first observe that the results are always the best for the same measure that was used as the optimization function. For example, if we want to minimize travel distance, the best result (34.7 km on average) would be achieved by using travel distance as the cost function in the clustering. The second observation is that the number of patients at risk can be improved significantly. With 90 min risk threshold, the number patients at risk reduces from 832 (5%) to 135 (1%) when optimizing for the patients at risk using the corresponding optimization function (Sigmoid). The difference is remarkable.

**Table 1:** Average travel time, distances, and the number of patients at risk when optimized for different cost functions

Optimization function	Euclidean distance	Travel distance	Travel time	Patients at risk
Original locations	29.0 km	36.6 km	35.3 min	832 (5%)
Euclidean distance	**27.9 km**	36.1 km	36.2 min	792 (5%)
Travel distance	28.4 km	**34.7 km**	34.1 min	519 (3%)
Travel time	29.8 km	36.8 km	**34.0 min**	488 (3%)
Patients at risk	44.3 km	54.9 km	48.6 min	**135 (1%)**

### Effect of the road network

**Fig. 8:**
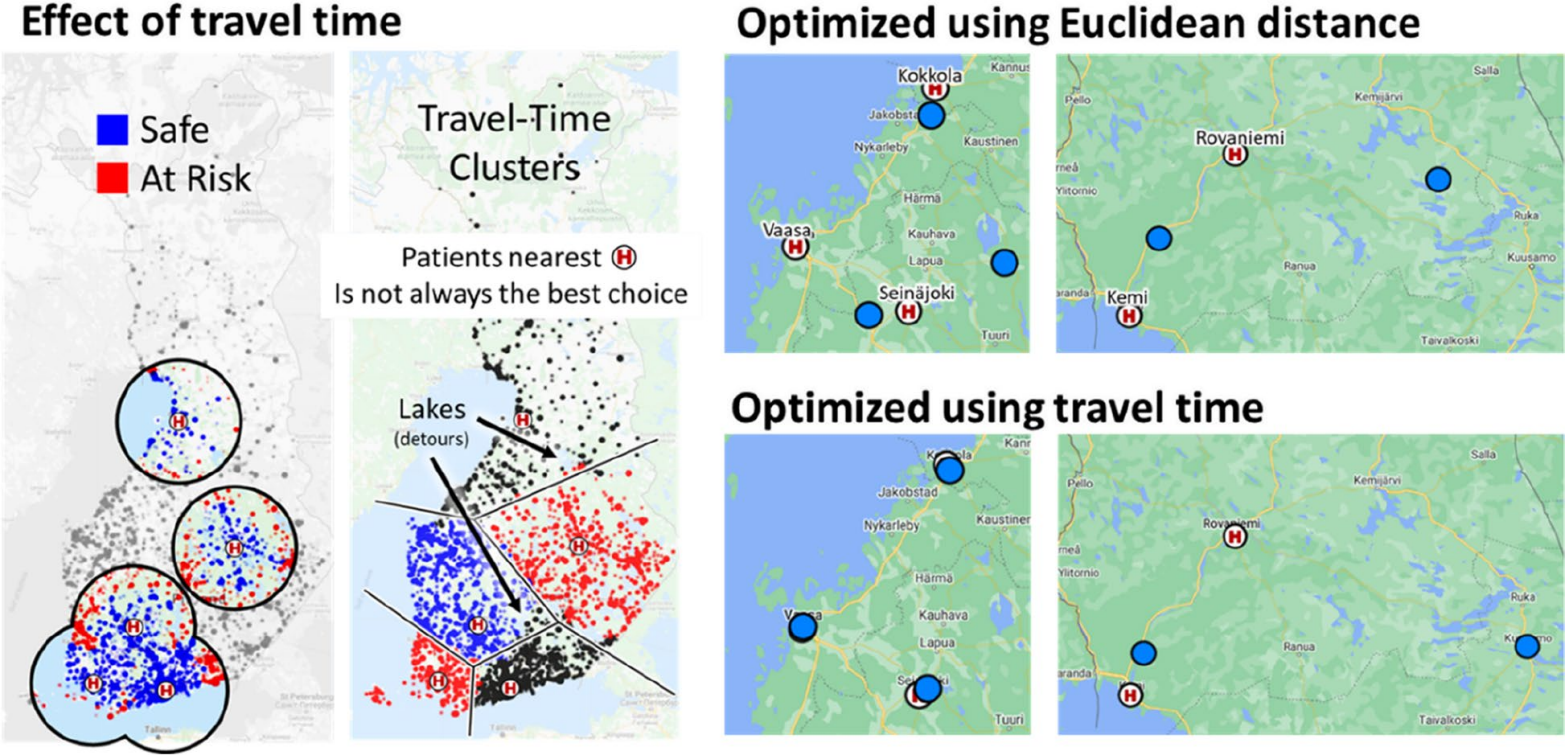
Accessibility of the hospitals depends on the road network. The division by the five university hospitals are presented by the black borders (Euclidean distance) and by colouring the patient locations (travel distance). They look quite similar but their effect on the optimization is significant

The effect of using road network is demonstrated further in Fig. 8. There are five university hospitals in Finland (Helsinki, Turku, Tampere, Kuopio, Oulu). If we map every patient to the nearest university hospital, we will get the division shown by the black lines if Euclidean distance is used. The difference when using travel distance seems marginal in this example but when applied in the optimization algorithm, the effect becomes significant. The optimized locations in many cases are close to their current locations in cities like Vaasa, Seinäjoki and Kokkola when the road network is used. However, with Euclidean distance hospitals would be located into arbitrarily places like Jumisko and Tervola in Lapland, or island in Alajärvi lake.

### Effect of optimization function

**Fig. 9:**
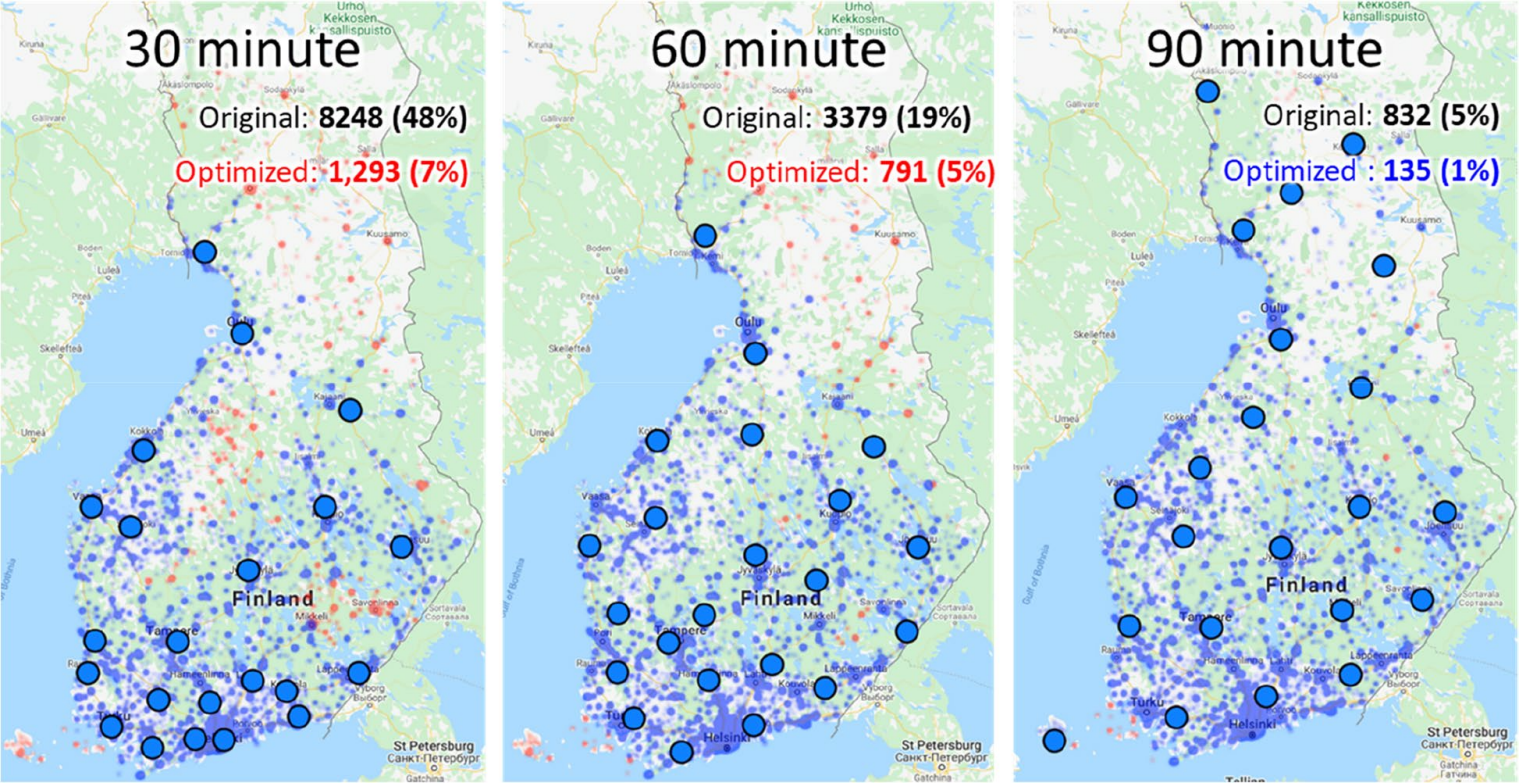
Results of the optimization with three different thresholds. The colours of the locations are red for the patients at risk when using 30 min (left), 60 min (middle) and 90 min (right) thresholds

Results when optimization for the patients at risk are further demonstrated in Fig. 9 with three risk thresholds (30 min, 60 min and 90 min). Reducing the risk threshold from 90 to 60 min would reach the same level (5%) as the current location with 90 min threshold. This means that the same number of people can reach the hospital in 1 h time with optimized locations compared to 1 h 30 min.

We can also see that that many optimized centre locations would move to sparsely population especially in North Finland. The reason is that most patients in south are safe when considering the higher 90 min threshold, which leads the optimization algorithm trying to catch the remaining patients at risk. This optimization, however, comes at a cost of increasing the average travel time of all patients. The optimization does not care if people need to travel 49 min (on average) instead of 35 min, as long as the time is below 90 min, see Table 1.

**Fig. 10:**
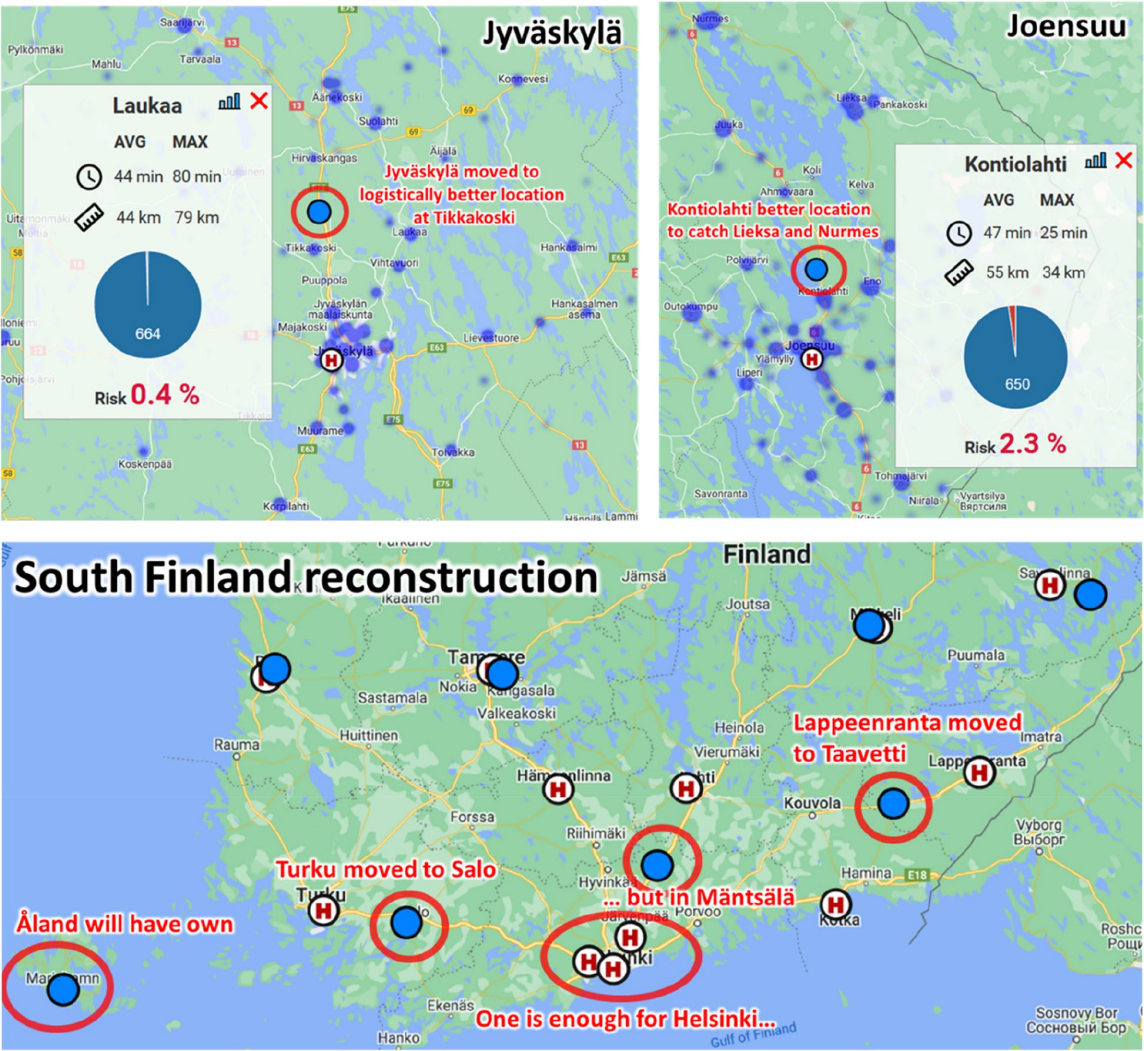
Optimizing patient at risk has many dubious side effects. Jyväskylä and Joensuu hospital would be moved to logistically better locations at Tikkakoski and Kontiolahti. South Finland reconstruction would leave only one hospital in Helsinki area and far away in Mäntsälä

Detailed effect in Central and South Finland are also demonstrated in Fig. 10 for the 90 min threshold. The optimization in Jyväskylä and Joensuu area are understandable from the logistics point of view, but the South Finland situation shows the effect of this optimization function. The optimized centre now in Mäntsälä is reached by *all* patients within 90 min. However, their original average travel time increases from 13, 22, 32 (Helsinki), 26 (Lahti) and 31 (Hämeenlinna) to 46 min (Mäntsälä). This does not make much sense. The results with 30 min threshold cause fewer radical changes but the patient at risk as such seems unsuitable cost function.

Travel distance or travel time as the optimization goal can also provide better locations and reduce those attributes, but the difference is less significant. Optimizing travel distance reduced the average distance from 36.6 km to 34.7 km (-5%), whereas optimizing travel time reduced average time from 35.3 min to 34.0 min (-3.7%). This would also reduce the number of patients at risk from 832 to 519 (when optimized for distance) and 488 (when optimized for time), respectively, and seems more suitable for patient at risk as well.

### How many hospitals?

**Fig. 11:**
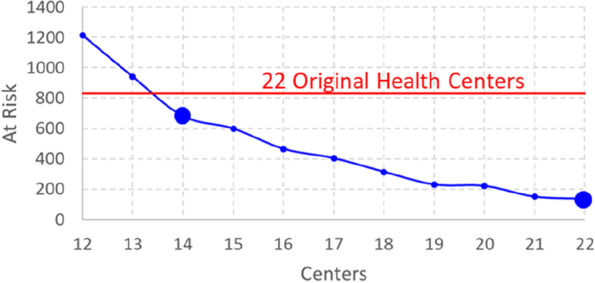
Effect of the number of hospitals when optimized for patients at risk (90 min)

Currently there is considerations to optimize the resources by cutting out some of the hospitals. We therefore study next how the reduction of the number of hospitals would affect the measures. Figure 11 shows that we could remove several hospitals without big change if the locations were optimized. We could still reach patients at risk at 1% level even if the number of hospitals was reduced from 22 to 19. Further reduction to 14 would still achieve better than the original 5% after which the proportion of patients at risk starts to increase radically.

**Fig. 12:**
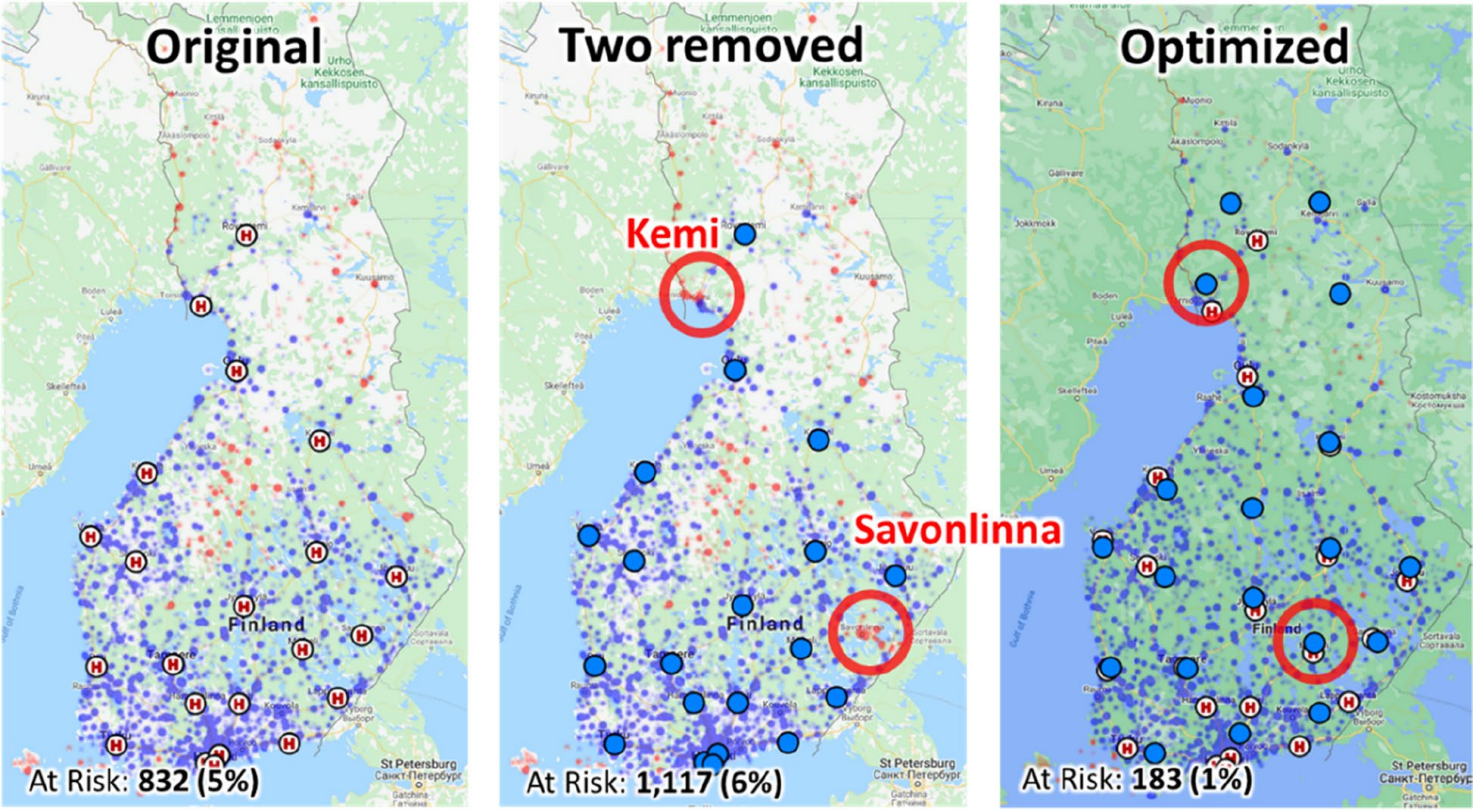
Effect of removing two hospitals (20 remaining). Savonlinna and Kemi (those most speculated to be axed) are removed in the middle. Re-optimizing the remaining 20 hospitals are shown on right

In real-life, we cannot relocate the hospitals just like that, and a more realistic question would be *which* PCI would be least harmful to cut-off? The two most speculated ones have been the two smallest, Savonlinna and Länsi-Pohja (Kemi), which urges us to test what would happen if they were removed. The results in Fig. 12 and Table 2 show their removal would have remarkable 20% increase in the patients at risk (from 832 to 1,117) which can be seen the red points in those regions. The algorithm would re-optimize the remaining 20 hospitals so that Savonlinna and Kemi would retain their own PCI units.

**Table 2:** The effect of removing two selected hospitals, and if only university hospitals were retained. Total patients: 17,346

			**Average travel:**	
**Test case**	**Hospitals**	**At risk**^**a**^	**Time**	**Distance**
All hospitals	22	832 (5%)	35 min	37 km
Two removed^b^	20	1,117 (6%)	37 min	39 km
Optimized *n* = 20	20	183 (1%)	46 min	51 km
University hospitals	5	7,332 (42%)	88 min	101 km
Optimized *n* = 5	5	5,023 (29%)	98 min	113 km

^a^Trip to nearest hospital > 90 min

^b^All hospitals except Savonlinna and Länsipohja

### Optimizing the travel time

**Fig. 13:**
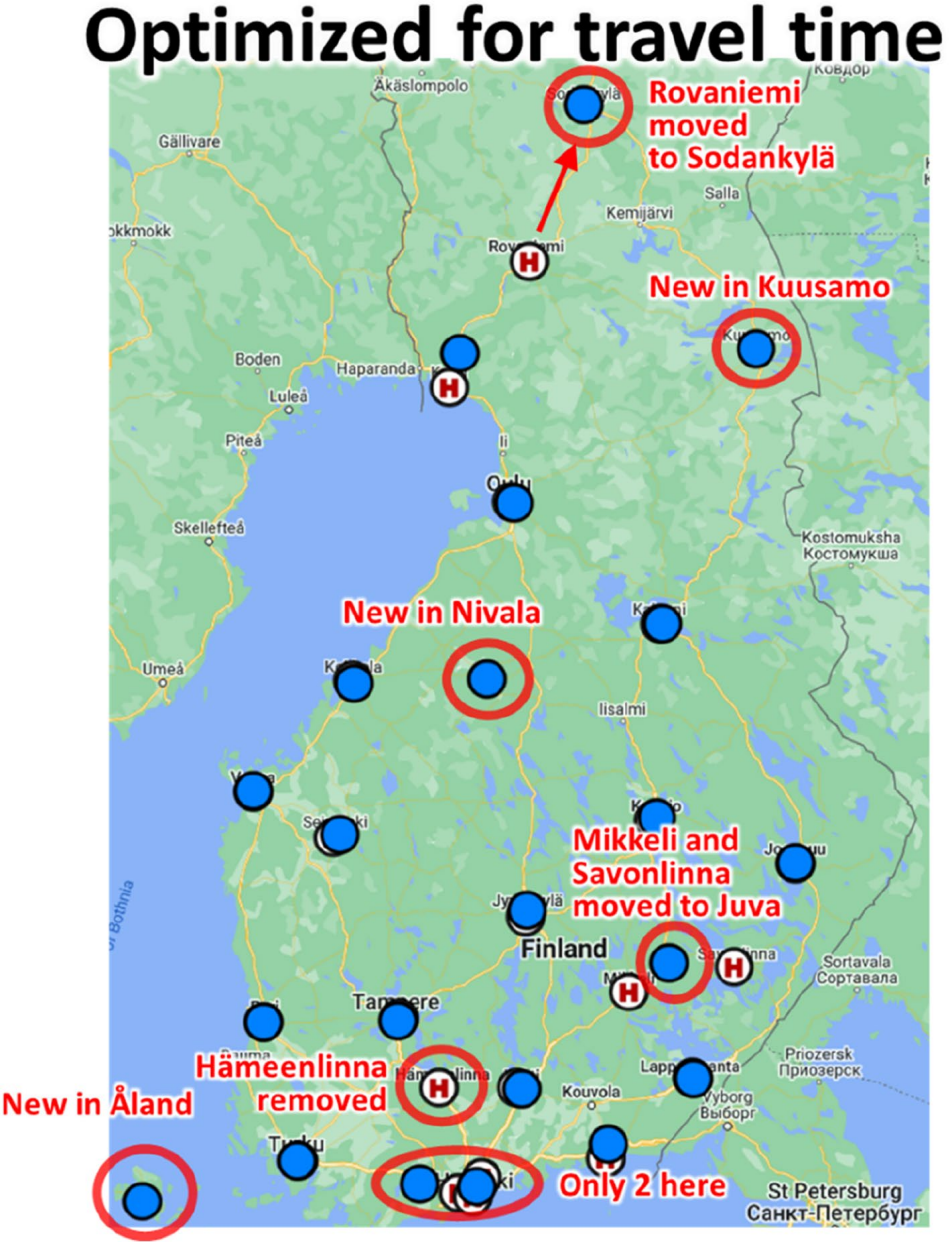
Optimization result using average travel time as the cost function

Optimizing for the travel time seems more meaningful than optimizing for patients at risk. Let us therefore study this optimization result in more detail. The optimized locations are shown in Fig. 13. The main observation is that most optimized locations are very close to the original locations. It shows that they are roughly at the places they should be, and well optimized from the STEMI patient point of view. The average travel time is 35 min and was reduced only by about 1 min by better optimization. The number of patients at high risk (90 min threshold) would be reduced more; from 5 to 3%.

The optimization algorithm kept most locations almost intact. Even logistics tuning in Jyväskylä and Joensuu would not happen when minimizing the average travel time instead of the patients at risk (targeting to reduce the worst cases). There are few changes the algorithm makes though as shown in Fig. 13.

1. Rovaniemi and Hämeenlinna are removed.
2. Capital area would have only two units instead of three, and one of them would be in Lohja.
3. The released resources would be allocated to Åland, Nivala and Kuusamo.
4. Mikkeli and Savonlinna would be merged and re-located in Juva in the middle.

The reason of putting one hospital in Åland is merely an artefact of it being island. The number of STEMI cases in the data was just 37. Their distance to Turku hospital is only 160 km but it takes about 6 h by ferry. These cumulate and causes a creation of a new PCI unit there. Turku hospital itself serves for 1,395 patients with an average distance of 28 km. In real-life, the few critically ill patients receive mostly care in Sweden due to the autonomous governance of the Åland islands and the proximity to Sweden.

Main result is that most hospitals are at excellent locations considering the STEMI patients already.

The other changes were caused by the optimization. The joint volume in Mikkeli (428) and Savonlinna (221) were about at the same as those of its surrounding hospitals Joensuu (602), Kuopio (787) and Jyväskylä (720). The merge is therefore as expected. Hämeenlinna (719) had similar volume but it is within < 1 h highway connection to Tampere, and likely therefore removed. New locations in Nivala (352) and in Kuusamo (123) are in areas without any major cities, and hospital, nearby. For minimizing the average travel time, the algorithm decided to allocate one PCI facility for both. It also considered Sodankylä more beneficial location for PCI unit than Rovaniemi due to long distances in the North.

Detailed statistics of the original and optimization locations are summarized in Tables 3 and 4. The current hospital locations in the Capital area follow the municipality borders as all three are in different municipality (Helsinki 13 min; Vantaa 22 min; Espoo 33 min). After optimization, only one remains in Helsinki (Tapaninvainio) roughly halfway between Helsinki downtown and Vantaa. The average travel time of patients to this location is clearly the shortest (22 min) among all optimized hospital locations. The second Capital hospital location is in Lohja (42 min). It covers large portion of the less dense areas from Hanko to Forssa, including also the westmost parts of Espoo (Finn).

**Table 3:** Statistics of the current hospitals

			**Average:**	
**Location:**	**Patients**	**At risk**	**Time**	**Distance**
Vantaa	1 511	0	22 min	23 km
Turku	1 485	70	42 min	37 km
Tampere	1 270	0	26 min	25 km
Helsinki	1 075	0	13 min	8,0 km
Lahti	1 043	0	26 min	25 km
Espoo	1 020	55	32 min	34 km
Pori	966	0	35 min	37 km
Seinäjoki	919	43	45 min	48 km
Kuopio	802	15	41 min	45 km
Jyväskylä	772	52	42 min	44 km
Oulu	761	83	42 min	46 km
Kokkola	750	65	44 min	50 km
Hämeenlinna	719	0	31 min	34 km
Kotka	682	0	35 min	37 km
Joensuu	672	70	39 min	42 km
Kajaani	596	151	60 min	73 km
Lappeenranta	493	0	23 min	24 km
Vaasa	455	0	29 min	29 km
Rovaniemi	443	219	90 min	104 km
Mikkeli	428	0	37 min	39 km
Kemi	263	9	22 min	26 km
Savonlinna	221	0	32 min	28 km
**TOTAL**	**17,346**	**832**	**35 min**	**37 km**

**Table 4:** Statistics of the new locations optimized for travel time

			**Average:**	
**Location:**	**Patients**	**At risk**	**Time**	**Distance**
Helsinki	3 271	0	22 min	23 km
Tampere	1 571	1	32 min	35 km
Turku	1 395	23	27 min	28 km
Lahti	1 210	0	26 min	29 km
Pori	984	3	35 min	38 km
Seinäjoki	892	4	43 min	44 km
Jyväskylä	752	34	41 min	44 km
Kuopio	727	24	37 min	41 km
Lohja	717	0	42 min	45 km
Joensuu	686	61	40 min	43 km
Kotka	685	0	30 min	32 km
Oulu	655	11	28 min	29 km
Juva	625	15	54 min	54 km
Kokkola	568	0	30 min	33 km
Lappeenranta	534	20	27 min	30 km
Kajaani	467	13	53 min	61 km
Vaasa	455	0	28 min	29 km
Keminmaa	447	84	57 min	61 km
Nivala	352	7	48 min	53 km
Sodankylä	189	114	102 min	115 km
Kuusamo	123	46	60 min	68 km
Åland	41	4	26 min	14 km
**TOTAL**	**17,346**	**464**	**34 min**	**36 km**

The volumes of the centres have somewhat changed due to the merges and by creating new centres in the sparsely populated areas. First, the volume of the smallest units in Kemi (263) and Savonlinna (221) have increased. The first one is moved slightly to North at Keminmaa (443) and covers now also Rovaniemi which increased its volume. The new centre at Juva (625) also has higher volume serving for both Savonlinna and Mikkeli. The three new centres are all small: Nivala (352), Sodankylä (189) and Kuusamo (123). From accessibility point of view, they are all well motivated but whether such low volumes would be economically feasible considering the overall health care services of the hospitals, is an open question.

## Discussion

We have analysed how optimizing hospital locations could improve patient access in diseases with critical time-dependency. To achieve this, we used Web tool designed to optimize spatial locations of PCI capable hospitals to promote timely access for STEMI patients. Three optimization goals were applied: 1) number of STEMI patients at risk to reside in areas with travel time of 90 min or over, 2) average travel distance, and 3) average travel time. For each of these goals, we observed the results of the optimized models against those of the current locations of PCI capable hospitals.

Our findings suggest that the network of PCI capable hospitals in Finland is rather well distributed along the country but could be still fine-tuned for better access. The results also suggest that possible closure of two smallest units (Savonlinna and Kemi) would increase the number of STEMI patients with travel time to PCI capable hospitals over the 90 min threshold by 34% (from 832 to 1,117).

Regarding optimization of travel times, the optimization result suggested several changes to the current hospital network. The removal of Hämeenlinna, the merging of two smaller units (Mikkeli and Savonlinna) into a new facility location at Juva, relocation of Rovaniemi to better serve the Northern parts. The optimization, however, did not consider the treatment capacity of the hospital and their emergency care services.

Accessibility may also be hampered furthermore by factors such as congested traffic or cultural factors causing delays in seeking care. Further studies should take into consideration factors such as regional variation of cardiovascular diseases and the population projections regarding ageing and internal migration. Despite considerable improvements in cardiovascular health, the population in Northern and Eastern parts of Finland still is burdened by these diseases more than the Southwestern parts.

The web tool provides a visual interface in which the effects of the abovementioned factors and different political decisions can be tested. The optimized models used in this study were restricted to a few selected parameter combinations but could be extended to consider more parameters such as lower and upper limits for the volume for the PCI units. One promising direction would be to optimize locations of other disease groups beyond the acute STEMI patients and finding disease combinations that would be most efficiently treated in the hospitals with similar service provision profiles.

The hospital network in Finland is predominantly publicly financed and its facilities constructed in the decades succeeding WWII as in other Western countries, but in early 2000s the network was considered outdated. Several efforts at centralization of these functions led to mergers and closures of secondary care facilities after 2013 [16]. However, the population projections show that further actions are needed as the number of elderly people is rapidly increasing, internal migration to larger cities continues, and shortage of health care workforce is common in sparsely populated regions. Specialized secondary and highly specialized tertiary care constitute the largest single cost component of the national health budget. Further consolidation of the hospital network would yield considerable returns but may be life-threatening in certain clinical conditions as the extended distances in the sparsely populated Finnish rural areas present considerable challenges.

Finland is not alone in the health care services reform as thorough restructuring of Danish hospital network began in 2007. This reform changed the hospital network structure of 40 public hospitals in 82 locations (2007) to 21 hospitals in 68 locations (2016) according to [17]. The restructuring took place in a democratic process subject to central guidelines and requirements including the central planning of specialties [18]. The permission to perform highly specialized treatments, such as PCI, is governed centrally. This reform has been deemed successful in terms of improved quality of care [19] due to stable costs and increased productivity [17].

Most of the studies on hospital network optimization have focused on geographic accessibility [20, 21]. These studies are prevalent also in the context of acute cardiovascular care [8] also in case of remote, sparsely populated regions [22, 23]. The Cardiac ARIA is a road time and distance based geographic model that determines what are the minimal services and resources required for the management of a cardiac event in any urban, rural, or remote population locations in Australia [22]. Some studies have focused on reorganizing their hospital network based on simulations on health care centralization [24] and some studies have aimed to reorganize the entire service system based on location-allocation models [25]. Huotari et al. have studied the Finnish hospital network based on accessibility if the number of maternity hospitals were reduced [26].

In Finland, municipalities with varying population base have been in principle responsible for organizing and financing health and social services for their residents [16]. Various attempts at health care and social welfare reform have been attempted in vain during the last two decades, but now a complete overhaul has been initiated and the new structures for organization and financing enters into force on Jan 1^st^, 2023. Currently no major plans have been initiated to further consolidate the hospital network, but as some of the new wellbeing services counties will most probably have difficulties in providing services for their residents, financial realities may necessitate these changes.

### Strengths and limitations

All the components of optimization have been carefully chosen based on the long-term experience of the authors in their respective research areas. Every single component from the clustering, travel time estimation and designing the cost function are well thought. The Web tool is also well designed, carefully programmed and reasonably flexible to allow testing new combinations if wanted. We have not seen anything similar developed elsewhere.

One limitation of the system is that the optimization algorithm was mainly tuned for the patients at risk criterion, which turned out to be not feasible. Some pre-optimized results were also run for other criterion with selected parameter combinations, but entirely new results cannot be currently run for the other criterion (travel time and distance) without some re-programming.

The study was limited to all the hospitals having a PCI unit even if only the five university hospitals have 24/7 capability. Pre-optimized results were run also for these but testing entirely new parameter combination is restricted to the same patients at risk criterion as above. Considering recent findings that differentiating STEMI from other types of myocardial infarction may be unreliable based on Finnish register data [27]. It is therefore possible that we were unable to identify all STEMIs – and that our study population may have also included some infarctions with less time-dependency (non-ST-elevation myocardial infarction I21.4 or unspecified myocardial infarction I21.9).

The travel time was estimated based on estimated road network distance without considering rush hours. More accurate result could be theoretically obtained by considering the time of the day in these calculations. However, we expect it would have just a minor fine-tuning effect due to the strong averaging effect of the optimization. One might also ask if the optimized locations would be different during rush hours. However, this bares very little relevance to the facility optimization unless someone plans the operations so that the PCI units could be dynamically re-located in certain times (rush hours). Until then, having heart-attack during the rush hours is just counted as bad luck.

The data selection included patients in Åland islands which is not even part of the Finland health care system; the patients were actually dealt in Stockholm (closer than Turku). The algorithm allocated unnecessarily one hospital to Åland, and effectively, removed one hospital from the mainland. The good side is that this data revealed how such artefact may affect the result and can be taken into account in future studies if such special situations are wanted to avoid.

The patient data have also other limitations. First, the postal code precision is a bit rough but good enough in this scenario as the errors from the estimation are minor compared to the 90-min hospital accessibility threshold. Second, the data represent the past demography in 2015–2018 and optimization might be better off using predicted future population. Clever predictor might even include factors like age and socio-economic status; or whichever factors increase the likelihood for heart attack.

## Conclusion

To answer the main question of the paper: yes, optimizing the locations of hospital by minimizing patients at risk can of course be done but we do not recommend doing so. It can theoretically reduce the patients at risk from 5 to 1% using the 90 min cutoff threshold. This itself is a good goal but it would also increase the average travel time of others. A somewhat better optimization function would be the average travel time itself as it would also reduce the number of patients at risk from 5 to 3% as a side result.

In real-life, we cannot simply re-locate hospitals into arbitrary locations. The web tool has demonstrated its power to provide important insight for the decision makers. It can reveal deficiencies of the current hospital network and indicate potential improvements. One main observation is that most hospitals are already well located for the STEMI patients’ point of view and only few were re-located by the optimization.

It seems that future attention should be targeted for better overall organization of the health care services rather than crude re-planning of their locations. STEMI cases are integrated into the other operations of hospitals and less likely to be operated as standalone units anywhere. The optimization of the entire healthcare system remains as a future goal but also a very challenging one. Nothing prevents to formulate it as an optimization problem as well, but it would be a very complex to define [28].

## Data Availability

The datasets analysed during the current study will be made publicly available in the UEF Machine Learning repository at http://cs.uef.fi/ml/impro/stemi/
